# Automated white matter fiber tract identification in patients with brain tumors

**DOI:** 10.1016/j.nicl.2016.11.023

**Published:** 2016-11-25

**Authors:** Lauren J. O’Donnell, Yannick Suter, Laura Rigolo, Pegah Kahali, Fan Zhang, Isaiah Norton, Angela Albi, Olutayo Olubiyi, Antonio Meola, Walid I. Essayed, Prashin Unadkat, Pelin Aksit Ciris, William M. Wells, Yogesh Rathi, Carl-Fredrik Westin, Alexandra J. Golby

**Affiliations:** a Brigham and Women's Hospital and Harvard Medical School, Boston, MA, USA; b Department of Biomedical Engineering, Akdeniz University, Antalya, Turkey; c Center for Mind/Brain Sciences (CIMEC), University of Trento, Rovereto, Italy; d Institute for Surgical Technology and Biomechanics, University of Bern, Switzerland

**Keywords:** Neurosurgery, Diffusion MRI, Tractography, Tumor, Fiber tract, White matter

## Abstract

We propose a method for the automated identification of key white matter fiber tracts for neurosurgical planning, and we apply the method in a retrospective study of 18 consecutive neurosurgical patients with brain tumors. Our method is designed to be relatively robust to challenges in neurosurgical tractography, which include peritumoral edema, displacement, and mass effect caused by mass lesions. The proposed method has two parts. First, we learn a data-driven white matter parcellation or fiber cluster atlas using groupwise registration and spectral clustering of multi-fiber tractography from healthy controls. Key fiber tract clusters are identified in the atlas. Next, patient-specific fiber tracts are automatically identified using tractography-based registration to the atlas and spectral embedding of patient tractography.

Results indicate good generalization of the data-driven atlas to patients: 80% of the 800 fiber clusters were identified in all 18 patients, and 94% of the 800 fiber clusters were found in 16 or more of the 18 patients. Automated subject-specific tract identification was evaluated by quantitative comparison to subject-specific motor and language functional MRI, focusing on the arcuate fasciculus (language) and corticospinal tracts (motor), which were identified in all patients. Results indicate good colocalization: 89 of 95, or 94%, of patient-specific language and motor activations were intersected by the corresponding identified tract. All patient-specific activations were within 3mm of the corresponding language or motor tract. Overall, our results indicate the potential of an automated method for identifying fiber tracts of interest for neurosurgical planning, even in patients with mass lesions.

## Highlights

• Spectral clustering machine learning approach for white matter tract identification
• Data-driven white matter parcellation learned from healthy subjects tractography
• White matter parcellation applied to 18 consecutive patients with brain tumors
• Arcuate fasciculus and corticospinal tracts identified in all patients
• All tracts within 3 mm of corresponding patient-specific functional activations

## 1 Introduction

Understanding of critical, individualized functional brain anatomy is necessary for neurosurgical planning. In neurosurgical patients, crucial areas to preserve during surgery include eloquent cortical regions such as sensory, motor, visual, and language areas, as well as related white matter connections or fiber tracts. Identification of these crucial brain areas using functional MRI (fMRI) and diffusion MRI (dMRI) has been shown to increase tumor resection, progression-free survival, and overall survival Petrella et al., 2006, Wu et al., 2007, indicating the important clinical potential of these presurgical MRI acquisitions. But the translation of fMRI and dMRI to widespread clinical use faces significant challenges, as discussed in recent reviews (Bullmore, 2012, Matthews et al., 2006, Rosen and Savoy, 2012, Ulmer et al., 2011).

In this paper, we focus on a particular challenge limiting the translation of dMRI to widespread clinical use: the need for expert processing and interpretation of dMRI tractography. Tractography data is complex, consisting of many hundreds of thousands of trajectories or “fibers” when seeded throughout the entire brain. In order to assess the patient-specific location of a particular fiber tract of interest, a trained expert must currently select the tract in an interactive way. The selection procedure requires the placement of multiple regions of interest in locations defined by the patient anatomy. This is time consuming, difficult to standardize across patients, produces variable results across operators and software packages Bürgel et al. (2009), and is complicated by the displacement of patient-specific brain anatomy due to mass effect. Furthermore, it is increasingly accepted (Caverzasi et al., 2015, Chen et al., 2015, Chen et al., 2016, Farquharson et al., 2013, Kuhnt et al., 2013, Mormina et al., 2015, Nimsky, 2014, Qazi et al., 2009) that for improved clinical anatomical accuracy, tractography must move beyond the standard diffusion tensor imaging (DTI) method, which can only represent one fiber at any location and is thus unable to model fiber crossing. Improved multi-fiber tractography methods, however, increase the difficulty of the expert selection procedure, requiring a higher number of regions of interest to restrict the selection. This is because these advanced multi-fiber tractography methods are able to trace a much higher number of fibers in any given region due to their increased sensitivity (Bastiani et al., 2012, Behrens et al., 2007, O’Donnell et al., 2013, Wilkins et al., 2015).

To aid processing and interpretation of complex, multi-fiber tractography data, we propose to perform atlas-based identification of key fiber tracts for neurosurgical planning. The goals of an automated method are to reduce the clinical time needed for human interaction and to increase the standardization of the presurgical plan. Increased standardization has the potential to avoid operator-dependent effects such as the choice of seeding or selection region, which are known to affect tractography results (Radmanesh et al., 2015).

Our overall approach is to leverage a database of data from healthy controls and to build models that are able to generalize to patients with mass lesions or displacement. In this work we extend and combine our methods for cluster-based (O’Donnell et al., 2006) automated data-driven tractography atlasing (O’Donnell and Westin, 2007) and tractography registration (O’Donnell et al., 2012) to create an end-to-end pipeline for automated analysis of neurosurgical patient data.

Our proposed method is designed to be relatively robust to challenges in neurosurgical tractography, which include peritumoral edema, displacement, and mass effect caused by mass lesions. We employ two-tensor unscented Kalman filter tractography (Malcolm et al., 2010), a multi-fiber tractography method that we have recently shown to be more sensitive than the clinical standard of single-tensor tractography in the presence of crossing fibers and edema (Chen et al., 2015, Chen et al., 2016). To identify tracts in a relatively robust way, despite displacement and mass effect, we use a strategy of large-scale features: major fiber tracts such as the arcuate fasciculus (AF) and corticospinal tract (CST) are quite large, traversing many image voxels, and have characteristic shapes and relationships to surrounding tracts. Such large anatomical features in the brain can potentially be identified in patients despite changes due to mass lesions, which can include displacement, infiltration, disruption, and peritumoral edema (Jellison et al., 2004). Our method uses the global interrelationships of the fiber tracts to aid identification: the fiber similarity between one fiber and many other fibers is used to perform spectral embedding of that fiber (O’Donnell and Westin, 2007). In this way, the feature vector describing a particular fiber is like a “fingerprint” that encodes its similarity to many other fiber tracts (not just to the nearest fibers).

In the rest of this paper, we first describe our proposed methods and then demonstrate their application to neurosurgical planning in a retrospective study of data from 18 consecutive neurosurgical patients with brain tumors.

## 2 Methods

Here we give a brief overview of our proposed pipeline, followed by a more detailed description of the datasets, computational processing methods, and experimental evaluations employed in this work.

### 2.1 Pipeline overview and methods background

Our approach has two main steps: learning a white matter parcellation and applying the parcellation to data from new subjects.

**Fig. 1:**
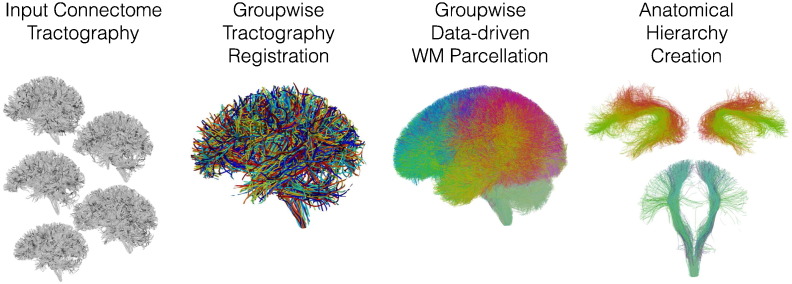
The overall pipeline for learning the data-driven white matter (WM) parcellation includes groupwise tractography registration, creation of a white matter parcellation (fiber cluster atlas) using groupwise spectral clustering of fibers, and visualization and organization of atlas clusters into an anatomical hierarchy using 3D Slicer. In the tractography registration, tracts from each subject are shown in a different color. In the white matter parcellation, colors are automatically generated from the spectral embedding, where each fiber cluster has a unique color, and similar clusters have similar colors.

First, our approach learns a model of the common white matter structures present in a group of healthy control subjects (Fig. 1) using groupwise tractography registration (O’Donnell et al., 2012) and clustering (O’Donnell and Westin, 2007). The unbiased entropy-based groupwise tractography registration method performs simultaneous joint registration of tractography in a group of subjects (O’Donnell et al., 2012) . Then the data-driven white matter atlas creation method employs group spectral clustering of tractography to discover structures corresponding to expected white matter anatomy. Bilateral clustering enables discovery of common structures across subjects and hemispheres (O’Donnell and Westin, 2007). These structures are represented as clusters in a “high-dimensional white matter atlas” in the space of the spectral embedding, which is created using the Nystrom method for analysis of large datasets (O’Donnell and Westin, 2007) . Finally, the fiber clusters are visualized and grouped by an expert to define structures of interest, which are stored in an *anatomical hierarchy*. Overall, this creates a data-driven white matter parcellation or *fiber cluster atlas* (Fig. 1). For more details, see Section 2.3.

**Fig. 2:**
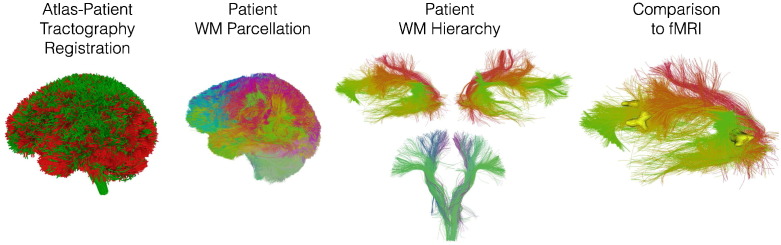
The pipeline for identification of key white matter (WM) tracts in patient data includes tractography registration, white matter parcellation via spectral embedding of fibers, and visualization of key patient-specific tracts using an anatomical hierarchy. In this study, patient-specific tracts are compared to patient-specific fMRI by computing distances to related functional activations.

Next, the fiber cluster atlas is used to automatically identify key patient-specific fiber tracts (Fig. 2). The entropy-based objective function (O’Donnell et al., 2012) is employed to register patient to atlas tractography. Then, automatic segmentation of patient tractography is performed by extending the spectral clustering solution, stored in the atlas, using the Nystrom method (O’Donnell and Westin, 2007) . The anatomical hierarchy is used to identify key patient-specific fiber clusters for visualization and comparison to fMRI (Fig. 1). For more details, see Section 2.4.

All software used in this project is publicly available as open source, including fiber tractography (Malcolm et al., 2010) (https://github.com/pnlbwh/ukftractography), computational tractography analysis methods (O’Donnell et al., 2012, O’Donnell and Westin, 2007) (https://github.com/SlicerDMRI/whitematteranalysis), and tractography visualization with anatomical hierarchies in 3D Slicer (Fedorov et al., 2012, Gering et al., 2001) (http://www.slicer.org) via the SlicerDMRI project (Talos et al., 2003) (https://github.com/SlicerDMRI).

### 2.2 Data acquisition and processing

Two datasets were used in this study: a healthy subjects dataset from the Human Connectome Project (HCP) (Essen et al., 2013) and a dataset of retrospective neurosurgical patient data.

#### 2.2.1 Human Connectome Project dataset

The dataset used to create the fiber cluster atlas consisted of 10 healthy subjects' data from the HCP^1^, processed following the HCP minimum processing pipeline (Glasser et al., 2013). HCP subjects were scanned at Washington University in St. Louis on a customized Siemens Skyra 3T scanner (Siemens AG, Erlangen, Germany) equipped with a standard 32-channel receive head coil and a “body” transmission coil (see Essen et al. (2013) for details). dMRI was acquired using a spin-echo planar imaging (EPI) sequence (TR  =  5520, TE  =  89.5, flip angle  =  78°, matrix  =  168 ×144, FOV=210 ×180  mm, 111 slices, voxel size  =  1.25  mm^3^), including 270 diffusion-weighted scans distributed equally over 3 shells of b  =  1000, 2000, and 3000  s/mm^2^ and 18 b  =  0 scans per subject. For this study, we extracted the b  =  3000 shell of 90 gradient directions and all b  =  0 scans for each subject. Angular resolution is better and more accurate at high b-values such as 3000 Descoteaux et al., 2007, Ning et al., 2015, and this single shell was chosen for reasonable computation time and memory use. DWIConvert (https://github.com/BRAINSia/BRAINSTools/) was applied during this preprocessing for data format conversion (NIFTI to NRRD).

#### 2.2.2 Neurosurgical patient dataset

For this retrospective study, we selected 18 consecutive patients (Table 1) with brain tumors who had diffusion MRI, functional MRI, T2-weighted, and contrast-enhanced T1-weighted images acquired presurgically. All imaging was acquired at Brigham and Women's Hospital on Siemens 3T scanners (Siemens Trio and Verio, Siemens Healthcare, Erlangen, Germany) equipped with a 12 channel head coil. DTI was acquired using an echo planar imaging (EPI) sequence (30 gradient directions, 1 baseline (b  =  0) image, b  =  2000  s/mm^2^, TR  =  12700, TE  =  98, flip angle  =  90°, matrix  =  100 ×90, FOV  =  22  cm, 59 axial slices, voxel size  =  2.3  mm^3^). Functional MRI images were acquired in the same session using EPI (24 contiguous axial slices, 5 mm slice thickness, TR  =  2000  ms, TE  =  30  ms, flip angle  =  85°, 64 ×64 matrix, voxel size  =  3.475 ×3.475 ×5 mm). fMRI was acquired as clinically indicated for each patient; tasks included block design motor (hand clench, toe wiggle, lip purse, finger tap) and language (antonym generation, sentence competition, auditory naming) paradigms and were presented using FDA approved hardware (goggles/headphones) and software (Nordic Aktiva, Nordic Neuro Labs, Bergen, Norway). High resolution anatomical T1 (with gadolinium contrast) and T2 weighted scans were acquired as clinically indicated for each patient. The study was approved by the Partners Healthcare Institutional Review Board, and informed consent was obtained from all participants prior to scanning.

##### fMRI processing

FDA-approved software was used for clinical fMRI analysis (BrainEx, Nordic Neuro Labs, Bergen, Norway). fMRI data were coregistered to the anatomical T2, motion corrected, smoothed, and analyzed using the general linear model. The resulting t score maps were independently thresholded by an expert and read by a neuroradiologist. The t score maps from the clinical report were imported into 3D Slicer, where an expert (PK,LR) selected the most appropriate activation for each task in order to exclude unrelated or noisy activations from comparison with fiber tracts. In each available language task, putative Broca's and/or Wernicke's areas were selected, while for each motor task, the relevant hand, foot, or lip activation was selected. fMRI language tasks commonly activate both hemispheres but are usually lateralized to the left hemisphere. If bilateral activations were present, putative Broca's and/or Wernicke's homologues were also selected in the right hemisphere. The thresholded and selected fMRI activations were exported as binary images and used to create surface models for comparison to fiber tracts.

##### dMRI processing

Diffusion Weighted Images (DWIs) were corrected for motion and eddy current distortions using DTIPrep (Oguz et al., 2014) (www.nitrc.org/projects/dtiprep). Images from all gradient directions were retained based on visual inspection of several patient datasets with an in-house tool indicating no gradients should be removed. Thus all 30 gradient directions were retained for analysis (Chen et al., 2015, Chen et al., 2016). We used 3D Slicer to obtain baseline images (B0, the b  =  0 image in the DWI volume) and a binary brain mask derived from the DWI images. A rigid registration was computed between the DWI baseline image and the T2 image. This rigid registration was later applied to the fiber tracts for visualization in 3D Slicer with anatomical T2 and fMRI.

#### 2.2.3 dMRI tractography

Whole-brain tractography of both datasets was conducted using a two-tensor unscented Kalman filter method (Chen et al., 2015, Chen et al., 2016, Malcolm et al., 2010) as follows. We used default values for UKF seeding and stopping fractional anisotropy (FA) thresholds, where these defaults have previously been empirically determined across multiple datasets (Chen et al., 2015, Chen et al., 2016, Malcolm et al., 2010). In UKF tractography, the FA seeding threshold refers to the FA of an initial single-tensor fit at the seed point, which is used to initialize the multi-fiber model. The FA stopping threshold pertains to tensor 1, the tensor that is tracked. An additional threshold, generalized anisotropy (GA), which is defined as a normalized variance of the diffusivities in all gradient directions, is used to assess the suitability of a multi-fiber fit for both seeding and stopping tractography. In combination, the FA and GA thresholds assess the suitability of the tensor being tracked and the overall signal.

*HCP dataset:* Tractography was seeded with 3 seeds per voxel, in all voxels within the binary brain mask where FA and GA were both greater than 0.18 (default). Tracking stopped where the FA value fell below 0.15 (default) or the GA fell below 0.09 (a value slightly below the 0.1 default, which was empirically determined to give good performance in HCP data).

*Patient dataset:* Tractography was seeded with 20 seeds per voxel (with larger voxels in the patient dataset than the HCP dataset, more seeds per voxel are needed) in all voxels within the binary brain mask where FA and GA were both greater than 0.18 (default). Tracking stopped where the FA value fell below 0.15 (default) or the GA fell below 0.075. The GA threshold was reduced below the default value in patient data to enable higher sensitivity for tracking in or near edema.

### 2.3 Creation of data-driven white matter parcellation (fiber cluster atlas)

Using the HCP dataset, we created a white matter parcellation in two main steps: data-driven groupwise analysis and expert creation of an anatomical hierarchy.

#### 2.3.1 Data-driven groupwise analysis

In this step, we learned a data-driven model of common white matter structures in the population. First, we computed an unbiased entropy-based groupwise tractography registration (O’Donnell et al., 2012) of all subjects in a multiscale fashion, first using affine transforms, then using a recently implemented extension to nonrigid b-spline transforms. Then we performed groupwise spectral embedding and clustering using the Nystrom method to identify common white matter structures in a data-driven way (O’Donnell and Westin, 2007). The spectral embedding creates a space that robustly represents the fiber tracts according to their similarities to all other fiber tracts. The Nystrom method uses random sampling to represent this space compactly while greatly reducing the number of fiber distance computations that must be performed. We note that many other methods for tractography registration, e.g. Durrleman et al., 2011, Garyfallidis et al., 2015, and clustering, e.g. Garyfallidis et al., 2012, Guevara et al., 2012 (Guevara et al., 2012, Guevara et al., 2011), Vercruysse et al., 2014, Wassermann et al., 2010, have been proposed (for a review on clustering, see O’Donnell et al. (2013)), so it is of future interest to test additional techniques in patient data.

We extended our groupwise clustering method to perform outlier removal by iteratively clustering and removing any outlier fibers that had low probability or affinity to their cluster. Fiber probability was defined based on pairwise fiber distances mapped through a Gaussian kernel (O’Donnell et al., 2012), which is the same as the fiber affinity we use for clustering (O’Donnell and Westin, 2007). At each iteration, for each cluster, the probability of each fiber was computed in a leave-one-out fashion using fibers from all other subjects in the cluster. Outlier fibers were rejected whose probability was more than two standard deviations away from the cluster mean probability, in a similar way to previously proposed spatial fiber outlier rejection (Guevara et al., 2011). In this way, uncommon tractography errors (spurious fibers) that were present in only one or few subjects were rejected from the atlas.

All parameter settings were determined empirically to enable registration and clustering of large multi-subject datasets, while keeping computational time and memory usage reasonable and feasible. It is well known that many tractography fibers are highly similar to their neighboring fibers (Presseau et al., 2015) , thus not all fibers are needed for analysis in order to learn common structures. Our overall strategy is to perform random sampling to reduce the number of fibers analyzed from each subject, keeping the total analyzed fibers (across subjects) sufficient to represent the anatomical structures of interest in the population. Empirically, we find that this total number of across-subjects fibers should generally be 100,000 or more for stable results. The number of fibers sampled from each subject is then calculated in order to reach the desired total number of fibers. In addition, fibers are thresholded by length: in the current project, a higher minimum length threshold (60 mm) for clustering avoided short-range connections of lower interest for neurosurgical planning. As the overall length distribution contained a majority of fibers below 60 mm, this length thresholding step reduced the overall dataset complexity and the number of clusters needed to describe the dataset. The choice of the number of clusters depends on the application, where a finer parcellation ( >1000 clusters) may be more useful for disease classification (Zhang et al., 2016), while for the current project, 800 clusters gave a fine parcellation that was still practical for expert visualization and grouping of clusters (see Section 2.3.2). We note that as datasets and tractography have improved, we have increased our default number of clusters from 200, which our previous experiments had indicated as the number of clusters that could be described in a single brain using single-tensor streamline tractography (O’Donnell and Westin, 2007).

Detailed parameter settings were as follows. The registration employed 20,000 fibers from each subject for a total of 200,000 fibers, with a minimum fiber length of 40  mm, and affine then coarse-to-fine b-spline registration with multiscale sigma values from 20 down to 2 mm and a final b-spline grid size of 8 × 8 × 8. The clustering employed 10,000 fibers from each subject for a total of 100,000 fibers, with a minimum fiber length of 60  mm, 800 clusters, 2500 fibers sampled for the Nystrom method, and two rounds of outlier rejection.

The clustering identified clusters bilaterally, which both improves clustering robustness (O’Donnell and Westin, 2007) and is useful for clinical visualization, in which neurosurgeons and neuroradiologists commonly use the technique of comparing tumor and contralateral healthy hemispheres to make clinical assessments. The final cluster atlas contained 800 structures that were consistently found across all ten healthy controls.

#### 2.3.2 Expert creation of anatomical hierarchy

The total number of clusters (800) was selected with the goal of separating all structures considered to be different anatomically. This fine subdivision allowed us to incorporate expert knowledge to combine clusters in order to to define an anatomically relevant tract for a particular application. For example, in our previous work (Voineskos et al., 2009, Whitford et al., 2010) we employed this strategy to combine multiple smaller clusters into larger regions that subdivided the corpus callosum (e.g. genu, temporal, occipital). In the current project, fiber clusters potentially related to motor and language functions were organized and grouped into larger anatomical structures of relevance for neurosurgery. This grouping was described in terms of an anatomical hierarchy, which can define multiple levels of structure subdivisions for anatomical visualization. Hierarchies are stored as part of the 3D Slicer scene file (in medical reality modeling language (MRML), an XML format), which can include multimodal medical images, surface models, tractography, and all relevant transforms relating the data for multimodal presurgical and intraoperative visualization. To enable the current study, we extended the existing anatomical hierarchy functionality in 3D Slicer 4.5 to support tractography data.

Experts (LJO, PK) viewed all clusters in the atlas to create the anatomical hierarchy, which was defined as follows. dMRI studies indicate that the corticospinal tract (CST), considered to be one of the most important pathways serving a number of motor functions essential for our voluntary movements (Al Masri, 2011), connects to primary motor, primary somatosensory, and dorsal premotor cortices, plus the supplementary motor area (Seo and Jang, 2013). However, for the purpose of neurosurgical planning, the CST of interest contains corticospinal and corticobulbar (face motor) fibers (Berman et al., 2004) and originates in primary motor cortex. Thus for this study, the CST hierarchy included clusters connecting precentral gyrus and brainstem. The arcuate fasciculus (AF) is possibly one of the most significant white matter fiber tracts related to language function (Dick and Tremblay, 2012). The AF hierarchy included all C-shaped fiber clusters connecting the temporal, parietal, and frontal lobes (known connections of AF Catani and Mesulam (2008)). The inferior fronto-occipital fasciculus (IFOF), inferior longitudinal fasciculus (ILF), and uncinate fasciculus (UF) are also thought to relate to language function (Chang et al., 2015, Duffau et al., 2009, Mandonnet et al., 2007, Martino et al., 2010, Papagno et al., 2010). The IFOF hierarchy included fiber clusters connecting frontal and occipital lobes (Catani et al., 2002) (additional clusters connecting frontal and parietal lobes were found, as expected in dissection studies (Martino et al., 2010), but were not included in the IFOF hierarchy for this initial retrospective study). The ILF (Catani et al., 2003, Mandonnet et al., 2007) hierarchy broadly included clusters connecting anterior temporal and occipital lobes. Finally, the UF (Von Der Heide et al., 2013) hierarchy included fiber clusters connecting frontal and temporal lobes in a hook shape.

### 2.4 Application of white matter cluster atlas to patient data

We applied the cluster atlas to parcellate the whole white matter of each patient's tractography data as follows. First, the entropy-based objective function used in the groupwise registration (O’Donnell et al., 2012) was employed to perform an affine registration of patient tractography to the atlas tractography. We note that in contrast to standard image-based atlas/patient registration, which is challenged by the presence of a tumor that does not exist in the atlas, tractography registration is relatively insensitive to the presence of a tumor, as there are generally very few fiber tracts traced in the tumor, and the objective function is relatively robust to any missing data.

After registration to the atlas, patient-specific fiber clusters were then detected bilaterally using spectral embedding of patient tractography, followed by assignment of each fiber to the closest cluster (O’Donnell and Westin, 2007). Outlier fibers were removed if their fiber probability/affinity given the atlas cluster was over 2 standard deviations from the cluster's mean fiber probability. The anatomical hierarchy was used to identify patient-specific key fiber tracts, and bilateral AF and CST clusters were then divided into right and left hemisphere structures to enable quantitative evaluation versus patient-specific fMRI in each hemisphere. All fiber clusters were then transformed from atlas space back to patient (DWI) space and then (rigidly) to patient T2/fMRI space for comparison to fMRI activations. For clarity, we note that the 800 clusters of each patient's white matter parcellation correspond directly to the 800 clusters in the atlas, such that the anatomical hierarchy from the cluster atlas is applied directly to each patient parcellation, regardless of the particular patient or coordinate system of the tracts. All transforms were applied to fiber tracts using 3D Slicer.

### 2.5 Quantitative evaluation

We used patient-specific motor and language fMRI to assess the success of atlas-based fiber clustering to identify key structures in tumor patients: the corticospinal tract (CST) and arcuate fasciculus (AF). CST tractography is known to connect to motor cortex electrical stimulation sites (Berman et al., 2004) and to motor fMRI (Archip et al., 2007) , and we have previously shown that two-tensor UKF is more sensitive than single-tensor streamline tractography to define tracts connecting to motor sites (Chen et al., 2016). Subcortical stimulation of AF causes language disruptions (Leclercq et al., 2010) , and AF tractography is expected to connect Broca's and Wernicke's fMRI activations (Vassal et al., 2016, Vernooij et al., 2007), though this may not always be the case with single-tensor tractography (Diehl et al., 2010). To perform quantitative evaluation, the signed distance was calculated from each fiber tract to the related fMRI activation(s). The signed distance is negative for tracts intersecting the fMRI and positive if no overlap occurs (Quammen et al., 2011). All available patient-specific language and motor fMRI activations were compared to the corresponding patient-specific AF or CST, and the minimum signed distances from each fiber tract to each corresponding fMRI surface model were recorded. The minimum signed distance indicated either the closest distance to the fMRI (if the distance was positive), or the maximum depth a fiber extended into the fMRI activation (if the distance was negative). Thus, a negative signed distance indicated that a fiber tract intersected the fMRI activation.

### 2.6 Comparison to expert tract selection

For an initial methods comparison, visual and quantitative comparisons to expert-selected tracts were performed in the first 9 patient datasets. Expert selection of CST and AF was performed by a trained neurosurgeon (AM) using regions of interest (ROIs) drawn on the T2-weighted image, with additional reference to the directionally-encoded color FA map. CST was selected with three inclusion ROIs at the level of the pyramid, the middle three-fifths of the midbrain, and the posterior limb of the internal capsule. For CST, a fourth large ROI was added to generally restrict CST to cortical areas of primary motor/M1, premotor, supplementary motor, and somatosensory cortex. AF was delineated with three ROIs as follows. On a coronal plane passing through the precentral gyrus, the first ROI encompassed the anteroposteriorly-oriented fibers adjacent to the lateral aspect of the CST, at the same level of the corpus callosum on a craniocaudal axis. The second ROI was created on an axial plane immediately above the level of the anterior commissure and included only the vertically-oriented fibers lateral to the atrium of the lateral ventricle. The third ROI was created on an axial plane, encompassing the anterolaterally-oriented fibers lateral to the lateral ventricle at the junction between the atrium and the temporal horn. All ROIs were placed bilaterally. Exclusion masks were used to avoid spurious fibers: each exclusion ROI was specific for each tract and hemisphere.

## 3 Results

### 3.1 Data-driven white matter parcellation in HCP dataset

**Fig. 3:**
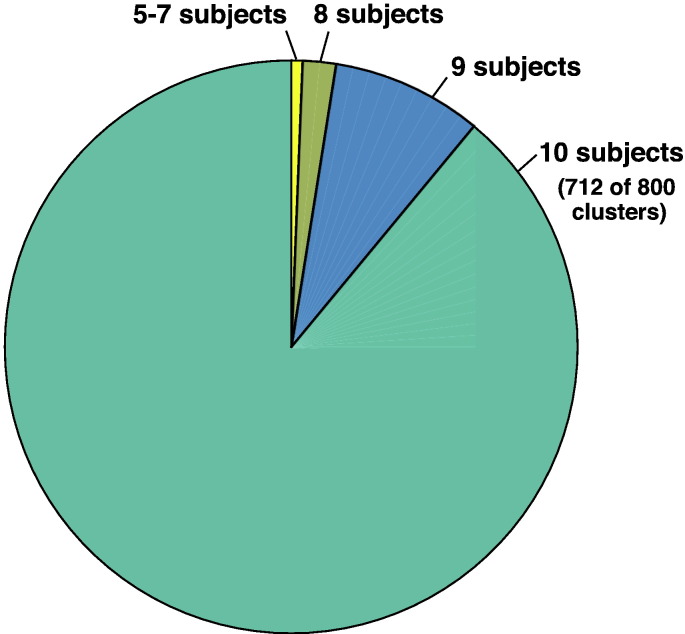
Data-driven white matter parcellation: cluster consistency across 10 HCP datasets. Of the 800 clusters, 712 (89%) are detected in all 10 subjects, and 780 (98%) are detected in at least 9 of 10 subjects. We note that this cluster consistency result is based on the 10,000 fibers that were randomly sampled from each subject for efficient groupwise clustering, meaning that on average there would be 12.5 fibers sampled per cluster per subject. Using a higher number of fibers per subject will increase this measure of cluster consistency (by increasing the number of clusters that can be detected in all 10 subjects), while increasing the computational run time.

The 800 fiber clusters were highly consistent across HCP subjects (Fig. 3). 89% of the clusters were detected in all subjects, and 98% of the clusters were detected in at least 9 of 10 subjects.

### 3.2 Identification of key tracts in fiber cluster atlas

**Fig. 4:**
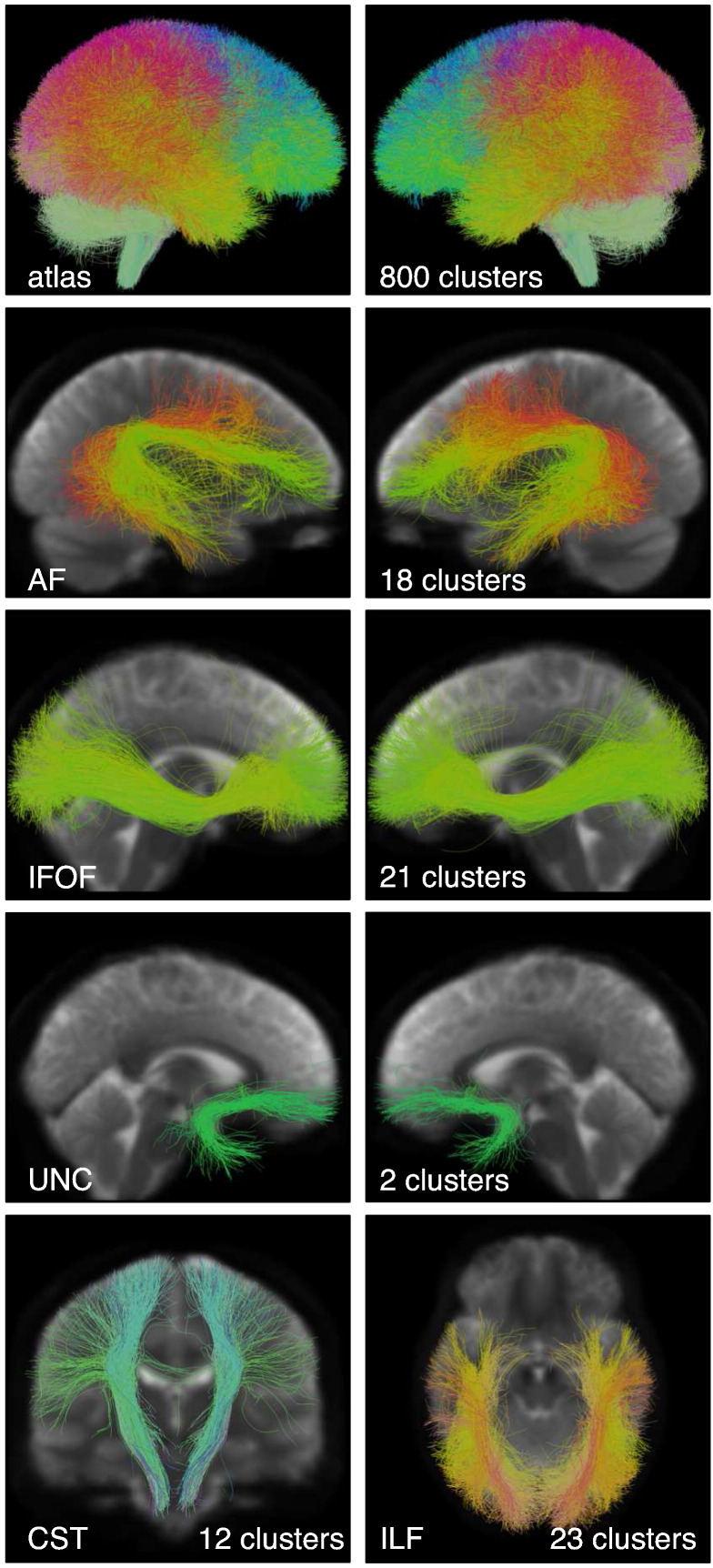
Creation of the fiber cluster atlas. Visualization of the data-driven white matter parcellation (top row) and the expert-defined anatomical hierarchies, which define structures of interest for neurosurgical planning. Note that each hierarchy is the union of several clusters. The number of clusters grouped into each hierarchy is shown. The image in the background is the average DWI baseline image from the ten subjects included in the atlas.

Anatomical hierarchies were created in 3D Slicer to organize the fiber clusters belonging to major tracts of interest (Fig. 4) supporting motor and language function. Note that multiple fiber clusters were included in each anatomical hierarchy.

### 3.3 Whole-brain white matter parcellation in patient dataset

**Fig. 5:**
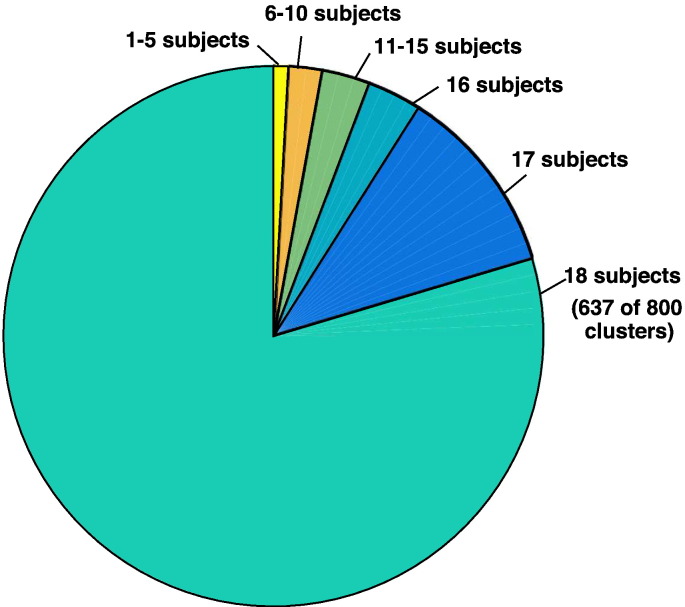
Cluster consistency across 18 neurosurgical patient datasets. Application of the cluster atlas to whole-brain tractography data from 18 patients indicates good generalization of the atlas to the patient dataset despite the presence of mass lesions. Of the 800 clusters, 637 (80%) are detected in all 18 patients, and 754 (94%) are detected in at least 16 of 18 patients. Note that clusters are found bilaterally, so this measure indicates the presence of the cluster in at least one hemisphere of each patient.

The fiber cluster atlas parcellates the entire white matter in each patient. Thus, in addition to our focus on main tracts relevant to neurosurgery, we were able to quantitatively evaluate the whole-brain parcellation of the patients. To give a measure of the generalization of the atlas to the patient dataset, we measured whether each cluster was present or absent in each patient (Fig. 5). This measure indicated robust generalization of the fiber cluster atlas to the patient datasets: 80% of the clusters were detected in all patients, and 94% of the clusters were detected in at least 16 of 18 patients.

### 3.4 Identification of key tracts in patient dataset

**Fig. 6:**
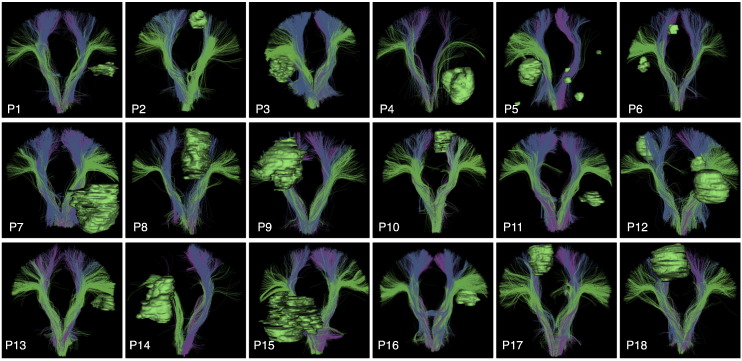
Automatically detected corticospinal tract clusters in all patient datasets (anterior view). Tumor surfaces are shown in green. Each cluster has a unique color, and similar clusters have similar colors. Multiple clusters are included in the corticospinal tract hierarchy, which groups putative corticospinal tract clusters for automated visualization.

**Fig. 7:**
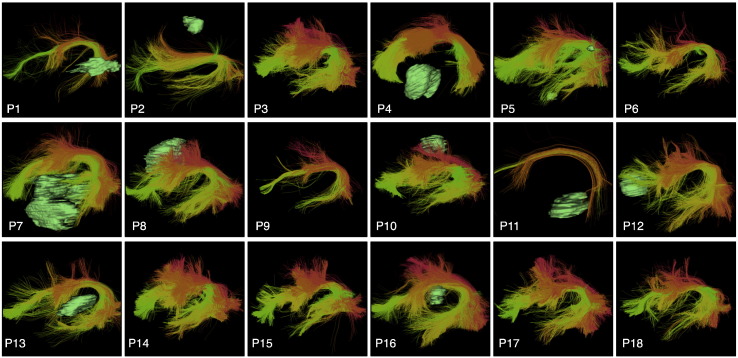
Automatically detected left arcuate fasciculus tract clusters in all patient datasets (view from left). Tumor surfaces are shown in green when they are near the tract. Each cluster has a unique color, and similar clusters have similar colors. Multiple clusters are included in the arcuate fasciculus tract hierarchy, which groups putative arcuate fasciculus clusters for automated visualization.

**Fig. 8:**
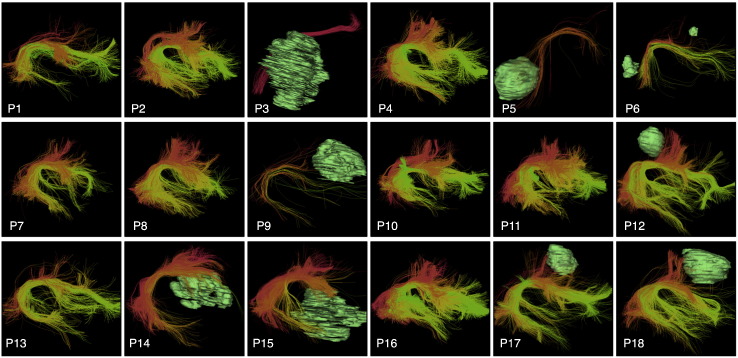
Automatically detected right arcuate fasciculus tract clusters in all patient datasets (view from right). Tumor surfaces are shown in green when they are near the tract. Each cluster has a unique color, and similar clusters have similar colors. Multiple clusters are included in the arcuate fasciculus tract hierarchy, which groups putative arcuate fasciculus clusters for automated visualization.

**Fig. 9:**
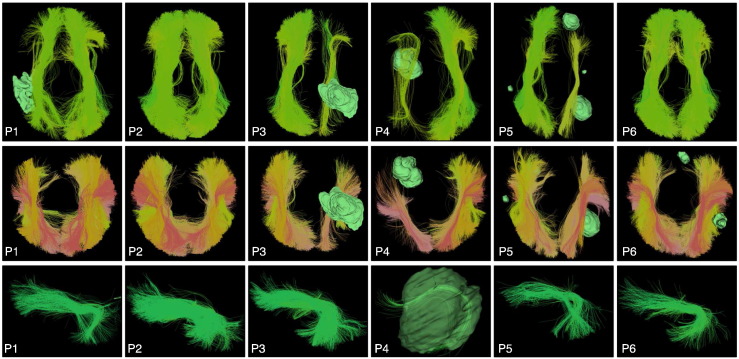
Automatically detected inferior fronto-occipital (IFOF, top row, superior view), occipito-temporal (ILF, middle row, superior view), and left uncinate (UF, bottom row, view from left) tract clusters, shown in the first six patient datasets. Tumor surfaces are shown in green when they are near the tract. Each cluster has a unique color, and similar clusters have similar colors. Multiple clusters are included in the tract hierarchies, which group putative IFOF, ILF, and UF clusters for automated visualization.

The left and right CST, AF, IFOF, UF, and ILF were successfully detected in all patient datasets. To facilitate visual assessment of performance across subjects, we rendered tracts (in the atlas coordinate system) against a black background (Fig. 6, Fig. 7, Fig. 8, and 9). We note that some detected tracts are apparently much smaller than others, and this is most apparent in the AF and IFOF results. The automated method identifies each fiber individually, based on its similarity to multiple fibers in the atlas, thus the method is not affected by the overall size of the fiber tract in an individual patient. This fiber tract size variability can be due to anatomical variability, tract lateralization (Catani et al., 2007, Lebel and Beaulieu, 2009, Propper et al., 2010), or the presence of a tumor and associated peritumoral edema.

### 3.5 Visual and quantitative evaluation of patient-specific key tracts using fMRI

**Fig. 10:**
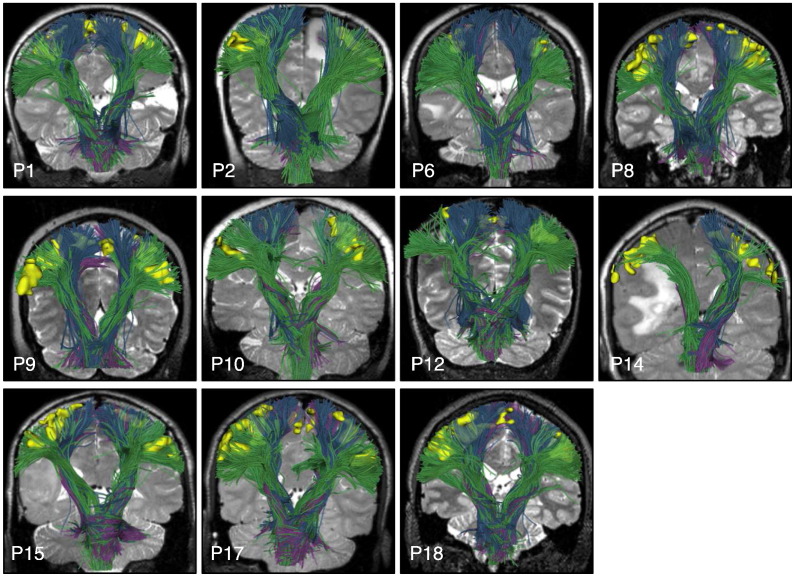
Automatically detected CST fiber tracts in patients with subject-specific task-based motor fMRI. Images show every patient-specific motor fMRI activation (yellow), with a T2-weighted image behind the fiber tracts, which are rendered partially transparent to better visualize the fMRI activations. All fMRI activations are intersected by CST fiber tracts except the right foot motor activation in the left hemisphere of P10 and the right hemisphere motor activations in P14.

**Fig. 11:**
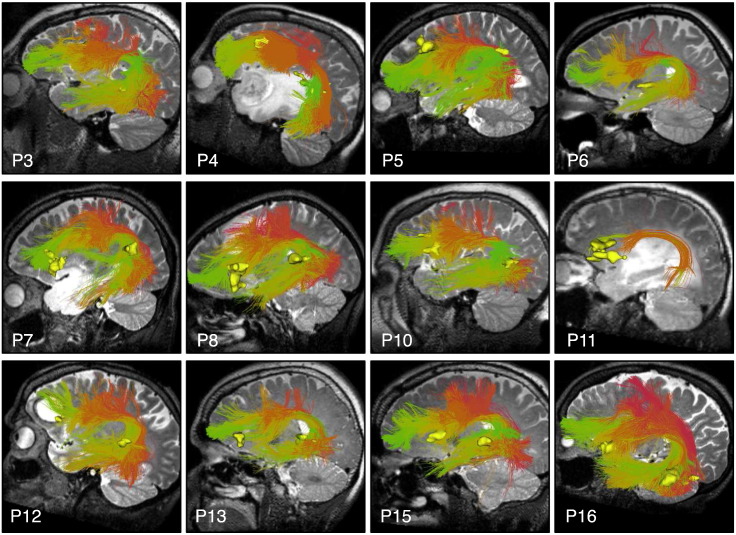
Automatically detected left AF fiber tracts in patients with subject-specific task-based language fMRI. Images show patient-specific language fMRI activations (yellow) in the left hemisphere, with a T2-weighted image behind the fiber tracts. All fMRI activations are intersected by AF.

**Fig. 12:**
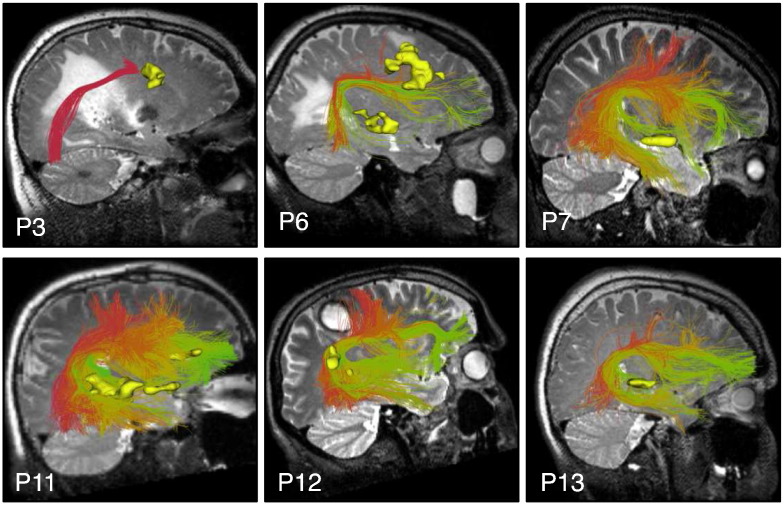
Automatically detected right AF fiber tracts in patients with bilateral language activations in subject-specific task-based language fMRI. Images show patient-specific language fMRI activations (yellow) in the right hemisphere, with a T2-weighted image behind the fiber tracts. All fMRI activations are intersected by right AF except putative Broca (P3 antonym task) and putative Wernicke (P6 audionaming task). Patients with language fMRI were right-handed except for P6, who had apparent right-hemispheric language lateralization according to fMRI.

**Fig. 13:**
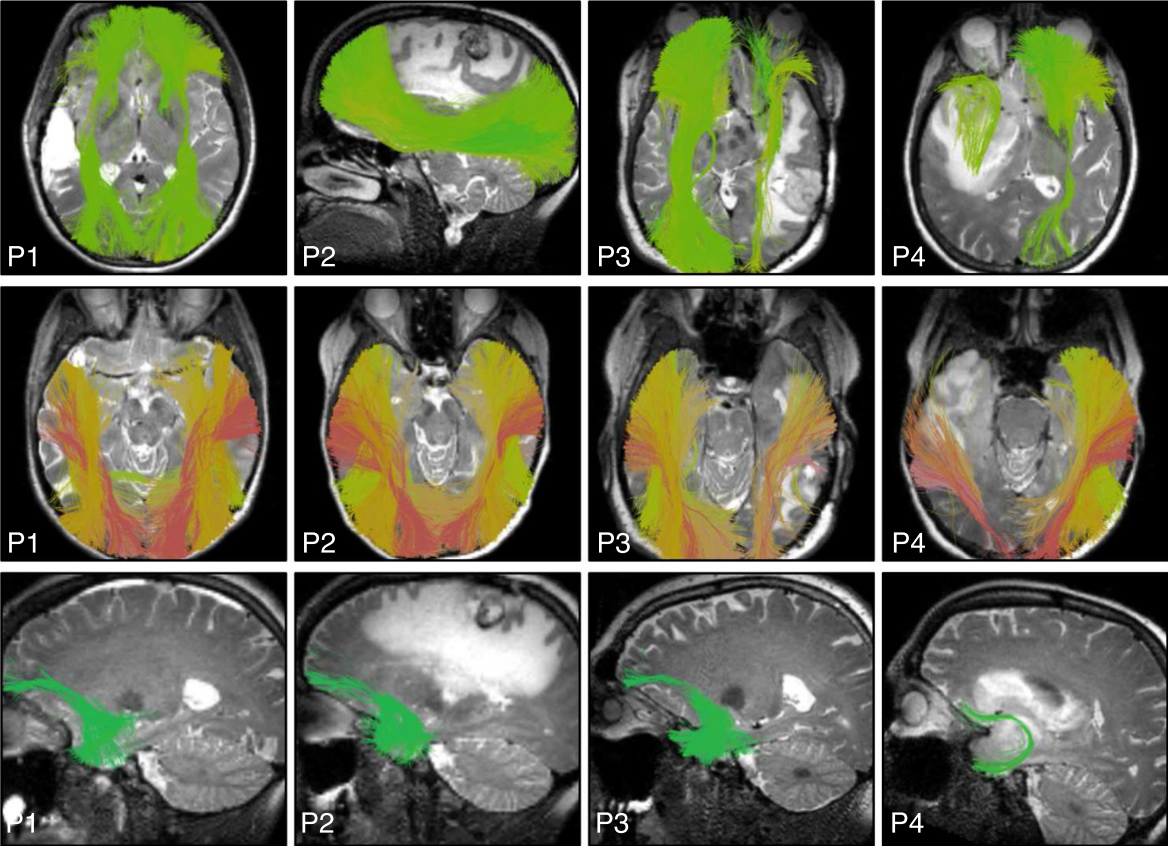
Automatically detected fiber tracts in the first four patient datasets illustrate example results in IFOF, ILF, and UF. A T2-weighted image is shown behind the fiber tracts.

**Fig. 14:**
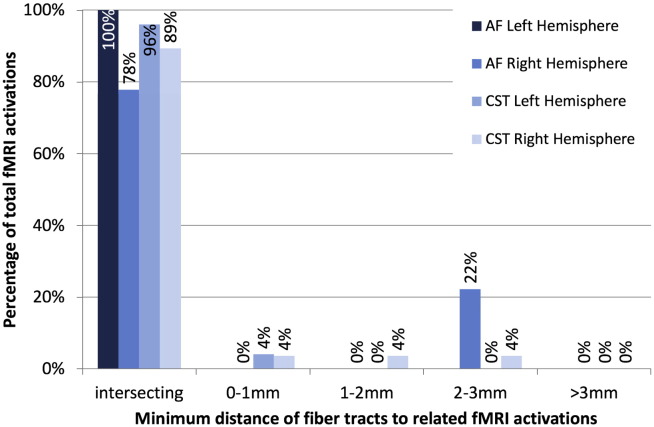
Quantitative results relating patient-specific automatically identified fiber tracts to patient-specific fMRI activations. Most fiber tracts intersect the related functional activations, and all are under 3mm from the related activations.

Finally, we assessed performance in all 18 patients by visualization of identified tracts versus patient-specific anatomical T2 images and fMRI (Fig. 10, Fig. 11, Fig. 12, and 13) and by quantitative comparison of AF and CST to patient-specific fMRI (Table 2, Fig. 14). Note that for each patient, some subset of the language tasks (antonym generation, sentence competition, auditory naming) and motor tasks (hand clench, lip purse, finger tap, toe wiggle) was acquired according to clinical considerations. Summary results regarding tract-fMRI intersection for each functional region are shown in Table 2, and quantitative distance results are summarized in Fig. 14.

In all 12 patients with language fMRI, the AF in the left hemisphere intersected all patient-specific activations. In the 6 patients with bilateral language fMRI activations, the right AF intersected at least one right hemisphere language activation in 5 patients, while the right AF intersected all right hemisphere language activations in 4 patients. In all 6 cases, the AF in the right hemisphere lay within 2.97 mm or less of the right-hemisphere language fMRI activations for all tasks.

Motor fMRI was acquired in 11 patients. The CST in the left hemisphere intersected at least one activation in all 11 patients, while the CST in the right hemisphere intersected at least one activation in 10 of 11 patients. For 10 of 11 patients, the left hemisphere CST intersected all patient-specific activations. The right hemisphere CST intersected all patient-specific activations in 10 of 11 patients as well. Across all patients, the greatest distance between the right hemisphere CST and patient-specific motor activations was 2.41  mm, while the greatest distance between the left hemisphere CST and patient-specific motor activations was 0.92  mm.

We also assessed the performance in tumor versus healthy hemispheres. The AF and CST intersected all patient-specific activations in all healthy hemispheres. In hemispheres with tumors, the AF and CST, respectively, were within 2.97 mm and 2.41 mm of all patient-specific activations.

### 3.6 Comparison to expert tract selection

**Fig. 15:**
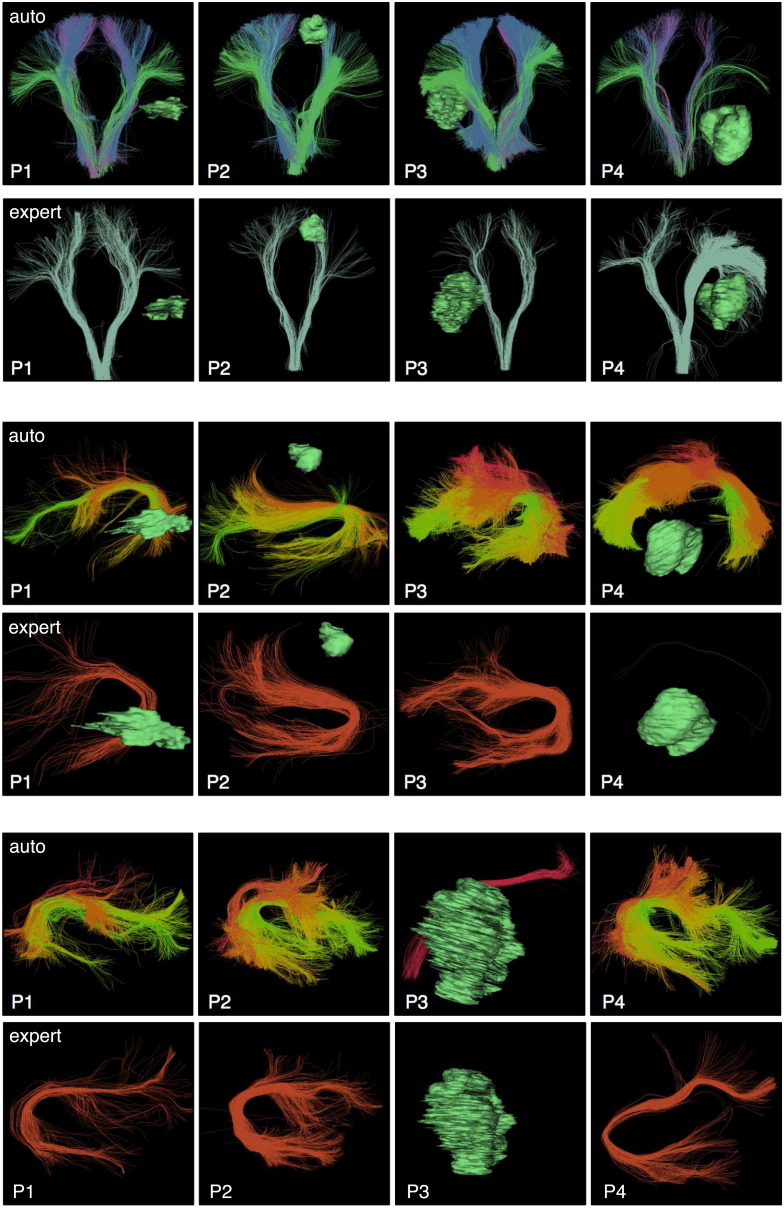
Comparison of expert tract selection versus automatic tract identification: visualizaton of results in the first 4 patient datasets in CST (top), left AF (middle), and right AF (bottom). In general, the automatic method tends to identify larger structures. All tracts were detected by both methods except for P3 right AF, which was not detected by the expert selection using anatomical ROIs. (Note that expert-selected left AF was detected in P4 but contains two fibers and is minimally visible.)

In the first 9 patient datasets, expert tract selection was performed. The expert-drawn ROIs identified left CST in 9 patients, right CST in 9 patients, left AF in 9 patients, and right AF in 8 patients (Fig. 15). Overall, the expert tract selection method produced tracts that were smaller in volume than the automatically identified fiber tracts (Table 3). Comparison to patient-specific fMRI indicated that expert-selected left CST was within 3.86 mm of all related activations, and expert-selected right CST intersected all activations. This performance in CST is similar to the automatic method, where all activations in both hemispheres were intersected in the first 9 patients. Expert-selected left AF was within 13.52 mm of all patient-specific left hemisphere activations, where 10 of 15 activations were intersected. In contrast, automatically identified left AF intersected all patient-specific language fMRI activations in the first 9 patients. Expert-selected right AF was identified in 2 of 3 patients with bilateral language activations: 1 activation was intersected, and expert-selected right AF was within 0.62 mm of all language activations in those 2 patients. In contrast, automatically identified right AF was found in all 3 patients: 2 activations were intersected, and automatically identified right AF was within 2.98 mm of all activations.

## 4 Discussion

In this paper, we have demonstrated high consistency across healthy and neurosurgical subjects in terms of the fiber clusters that may be defined using unscented Kalman filter two-tensor tractography. The automatic patient-specific tract identification was assessed as having very good colocalization with patient-specific fMRI activations. However, tractography methods are under active development (Jeurissen et al., 2014, Reisert et al., 2011, Tournier et al., 2012) and evaluation (Bucci et al., 2013, Fillard et al., 2011, Mormina et al., 2015, Neher et al., 2015, Pujol et al., 2015, Thomas et al., 2014), with many competing algorithms to choose from, and there remains significant anatomical controversy about the true extent and termination of many fiber tracts in the human brain (Dick and Tremblay, 2012, Meola et al., 2015, Von Der Heide et al., 2013) . Thus, there is unavoidable uncertainty in these and any other tractography results. Overall, the assessment of which tractography method, or which combination of dMRI acquisition strategy and tractography method, performs the best for neurosurgical planning remains an open question for future research.

Two well known issues in tractography are false positive (anatomically incorrrect) and false negative (missing fibers) errors (Catani, 2007, O’Donnell and Westin, 2011). We believe we have ameliorated the false positive issue to a certain extent by rejecting outlier fibers that were improbable, given the other subjects, during the process of atlas creation and again during patient tractography identification. But with our approach, any false positive errors that are strongly present across subjects (i.e. errors typically made by the tractography method) would still be consistently represented in the atlas. Other approaches have been proposed to filter tractography (Smith et al., 2013, Smith et al., 2015), which operate at the single-subject level and could be tested in the context of neurosurgical planning. In the context of our current method, reducing false positive fibers using stricter outlier removal might not make sense, because high sensitivity is desirable for neurosurgical planning. In fact, an error in one spot along the fiber does not necessarily indicate that the rest of the fiber is false. This is because decisions are made locally during tracking in UKF tractography and in the majority of tractography methods used for neurosurgical planning.

The second tractography issue is false negative fibers due, for example, to fiber crossings. Clinically, this is especially problematic because tractography (especially that based on single-fiber diffusion tensor imaging or DTI) has difficulty tracing the the lateral projections of the CST, which cross the AF and superior longitudinal fasciculus (Behrens et al., 2007, Berman et al., 2004, Jones, 2008). We and others have shown that multi-fiber tractography methods are important to improve the anatomical accuracy of AF and CST for neurosurgical planning (Bucci et al., 2013, Caverzasi et al., 2015, Chen et al., 2015, Chen et al., 2016, Farquharson et al., 2013, Kuhnt et al., 2013, Mormina et al., 2015, Nimsky, 2014, Qazi et al., 2009). The problem of false negative or missing fibers was reduced in the current study by using a sensitive multi-fiber tractography method; however, it is not possible to assess the existence of false negatives without ground truth. Furthermore, the fact that fiber clusters are found bilaterally in our method allows comparison across hemispheres, and it is visually apparent in several patient datasets that the size of the tract that can be identified in the tumor hemisphere is smaller than that in the contralateral hemisphere. While some lateralization is expected, for example in AF (Catani et al., 2007, Lebel and Beaulieu, 2009, Propper et al., 2010), tract lateralization in tumor patients is a challenge because it is not possible to know whether apparently “missing” tracts are actually destroyed, or whether they are affected enough by edema and/or infiltration to prevent tractography. Though we have previously shown that UKF two-tensor tractography can trace a larger AF and CST than single-tensor streamline tractography in the presence of peritumoral edema (Chen et al., 2015, Chen et al., 2016), tractography in edematous regions is clearly still a challenge. In such cases, additional interactive tractography (Golby et al., 2011) in the region of the detected fiber tracts, as well as correlation with fMRI, could help provide more information on a patient-specific basis.

The currently presented results indicate a very sensitive detection of key fiber tracts, especially AF. The patient-specific structure-function colocalization in the current study, where the AF in the left hemisphere intersected the left hemisphere language fMRI activations for all tasks, is higher than the structure-function colocalization reported in previous studies that relied on the single diffusion tensor (DTI) model to perform tractography of AF. For example, in one study that compared DTI tractography of AF to implanted electrode location in epilepsy, good colocalization of AF (defined as within 10 mm of the functional region) was only found for 84% of electrodes in Broca's area and 56% of electrodes in Wernicke's area (Diehl et al., 2010). In another study, anterior terminations of AF were found primarily in premotor cortex, not in Broca’ area, using DTI tractography (Bernal and Ardila, 2009).

In related work, Tunc et al. have recently proposed a method for automated identification of fiber tracts in presurgical planning (Tunç et al., 2015). Their work also adopted the strategy of building a model or atlas by clustering tractography from multiple subjects, an approach first reported by our group (O’Donnell and Westin, 2007). Their clustering method relied on a “connectivity-based” strategy, in which fibers were clustered according to the pattern of local cortical connectivity of each voxel along a fiber (Tunç et al., 2015). This is a promising strategy, but it requires parcellation of the patient cortex via image registration, a potentially difficult approach due to the high inter-subject variability of cortical topography (Desikan et al., 2006) and the known challenges in image registration due to the presence of a tumor (Kaus et al., 2001, Risholm et al., 2010). In contrast to Tunç et al. (2015), our proposed method does not rely on an image-based registration or a cortical parcellation. Instead it employs overall fiber shape and location, measured using pairwise fiber similarity, both for registration and spectral clustering. Another important difference relative to Tunç et al. (2015) is that we applied a multi-fiber tractography approach (not single-tensor DTI tractography), which provides improved anatomical accuracy in neurosurgical planning (Caverzasi et al., 2015, Chen et al., 2015, Chen et al., 2016, Farquharson et al., 2013, Kuhnt et al., 2013, Mormina et al., 2015, Nimsky, 2014, Qazi et al., 2009).

In our study, 94% of the 800 fiber clusters were found in a very robust way (in 16 or more of the 18 patients), when clustering densely seeded whole-brain tractography. Due to differences in acquisition, it was expected *a priori* that the clinical dMRI scan would be less sensitive in terms of the number of anatomical structures that could be detected relative to the advanced HCP acquisition, in which 98% of the 800 fiber clusters were identified in at least 9 of 10 subjects. This 98% measure in healthy control HCP data is actually an underestimate of the cluster consistency, as it is based on downsampled tractography data from each subject to enable efficient groupwise clustering. We believe that using HCP data improves the overall quality of our data-driven parcellation; however we have previously successfully applied the clustering method to create atlases using more standard acquisitions (O’Donnell et al., 2009, Propper et al., 2010, Whitford et al., 2010) . Our experience indicates that the data used to learn the atlas should be at least as good or better than the data that will be segmented using the atlas, that the tractography method should be the same for both datasets, and that bilateral clustering improves robustness (O’Donnell and Westin, 2007). We have also observed that using multi-fiber tractography increases the consistency of the clusters found across subjects. In the future, improved dMRI scans with higher b-values and/or multishell data, as well as improved tractography methods and better modeling of edema, are expected to increase the quality of patient tractography, bringing it even closer to the quality of the HCP dataset.

We performed an initial comparison to expert-selected tracts in the first 9 patients. This experiment serves mainly as a proof of concept that automated tract identification can have comparable performance to expert tract identification. Tract selection results are known to vary across expert raters (Bürgel et al., 2009), where variability can occur in several situations, such as deciding on the size of the ROI, the number of ROIs drawn, and the slice(s) on which the ROIs are drawn (Voineskos et al., 2009). Thus a more complete clinical evaluation including additional raters and patients is a clear avenue for future work. However, in this initial comparison there were clear trends demonstrating that the automatic method identified larger structures that intersected more fMRI activations. This comparison result reflected the difference in how ROIs were defined by the expert versus how clusters were included in the tract hierarchies. For example, the expert method was much more specific in the brainstem where two CST ROIs were placed. In contrast, the automatic method visually demonstrated more fibers connecting to lateral motor regions (e.g. face and hand), as the CST hierarchy was defined primarily according to connection to motor cortex. In AF, the automatic method was apparently less sensitive to tract displacement due to mass effect, demonstrating larger AF volume across patients. We tested expert tract selection using segmented ROIs, and another option is interactive selection using boxes in 3D, which tends to be more inclusive as the user has less specific control over the shape of the ROI. ROI placement strategies may also be affected by an expert's familiarity with, or preference for, single-fiber versus multi-fiber tractography, where the latter method is known to generate more connections and have higher sensitivity (Bastiani et al., 2012, Behrens et al., 2007, Wilkins et al., 2015) with larger tract volumes (Chen et al., 2015, Chen et al., 2016).

Our data-driven tractography parcellation achieves a fine subdivision of the white matter with the potential to be specific to tracts of interest. For example, our approach naturally subdivides the arcuate fasciculus/superior longitudinal fasciculus complex into multiple parts as expected from neuroanatomical research (Catani and Jones, 2005, Makris et al., 2005, Martino et al., 2013). AF subdivisions are shown in Fig. 4, Fig. 7, and 8. SLF was also divided into multiple subdivisions by the clustering method (not shown). We note that the AF and CST tract hierarchies of interest included 30 of the total 800 clusters that were defined using our approach. The cortical regions involved in the language network are widely distributed (Corina et al., 2010, Huth et al., 2016) , and we have not yet evaluated additional tracts related to language function (Chang et al., 2015), such as the superior longitudinal fasciculus (Makris et al., 2005, Martino et al., 2013), frontal aslant tract (Catani et al., 2012, Catani et al., 2013), middle longitudinal fasciculus (de Champfleur et al., 2013, Makris et al., 2009) , and extreme capsule (Makris and Pandya, 2009).

One limitation of our method is that it is difficult, given our current technology, to separate motor from sensory fibers in the CST hierarchy. In fact, it is not completely clear if this separation is possible based only on tractography information, because approximately two-thirds of corticospinal fibers have been shown to originate from axons of pyramidal cells, mainly from primary motor/M1 (Brodmann's area 4), premotor and supplementary motor (Brodmann's area 6); while the remaining one-third of fibers has been shown to arise from the somatosensory cortex (Brodmann's areas 3, 1 and 2) (Mendoza and Foundas, 2007, Snell, 2010, Stippich, 2007). For the purposes of this study, any fiber clusters in the atlas that primarily connected to postcentral gyrus or to regions anterior of the precentral gyrus were not included in the M1 CST hierarchy. The results in this study indicate good sensitivity of CST detection, based on good colocalization with patient-specific fMRI activations. However, to potentially increase specificity of motor fiber identification, future work could take into account additional information such as multimodal imagery, cortical geometry, and functional information when creating and applying the white matter parcellation. Robustly tracing the CST from cortex to brainstem is still a challenge, and improvements in data preprocessing for distortion correction may improve depiction of the corticospinal tract and brainstem region in HCP datasets (Irfanoglu et al., 2015).

There are some additional limitations of the current study. One limitation of our method is the run time (approximately 2.5  h per patient dataset to seed, register, and cluster all fiber tracts using 20 processors for tractography and 2 processors for the rest of the pipeline) and the fact that some of the steps, as currently implemented, require knowledge of the command line, limiting their ease of use for clinically-trained personnel. We are currently investigating an optimized implementation for improved useability. In this project, image distortions caused by eddy currents and/or motion were corrected in the traditional way by registration to the baseline image (Graham et al., 2016, Oguz et al., 2014) such that the DWIs were considered to be in the space of the relatively undistorted baseline image, which was then rigidly registered to the T2 space. This does not correct for echo-planar imaging distortions, which are expected to be on the order of 2 mm in the phase-encode direction in patient data (Treiber et al., 2016), and are generally neglected in clinical practice. Newly-proposed methods (Andersson and Sotiropoulos, 2016, Irfanoglu et al., 2015) do have high potential for correcting DWI distortions in the future. Finally, potential limitations of the proposed method may be patient-specific, as some tumors will affect tracts more than others. In the future, an interactive system could allow the clinician to both increase and decrease the number of fibers included in the tract of interest, for example by expanding and contracting the region of the spectral embedding space considered to belong to the tract. This type of visualization could provide practical information about the uncertainty of automated tract detection.

Overall, the results of our study indicate the high potential of an automated method for identifying fiber tracts of interest for neurosurgical planning. We note that this study also supports the utility of our methods and open-source tools for other applications of white matter parcellation (O’Donnell et al., 2013), such as our recent work in autism (Zhang et al., 2016). In the current study, we evaluated a limited number of selected fiber tracts considered to be of importance for neurosurgery, and we demonstrated their robust identification across patients using multi-fiber tractography. We plan further multi-modal data-driven investigation of the relationship of patient-specific whole-brain fiber clusters to patient-specific fMRI in order to more finely define which fiber clusters may be of greatest functional importance for neurosurgical planning.

## Figures and Tables

**Table 1:** Patient demographic data and pathology. W.H.O.: World Health Organization.

	Patient information		
Patient	Age	Gender	Tumor type
P1	28	F	Oligodendrioma, W.H.O. grade II
P2	34	F	Recurrent metastatic carcinoma, lung primary
P3	57	M	Glioblastoma (GBM), W.H.O. Grade IV
P4	66	F	Glioblastoma (GBM), W.H.O. Grade IV
P5	63	M	Metastatic melanoma
P6	52	F	Metastatic carcinoma, breast primary
P7	70	M	Anaplastic astrocytoma, W.H.O. Grade III
P8	26	F	Anaplastic astrocytoma, W.H.O. Grade III
P9	57	F	Diffuse astrocytoma W.H.O. grade II
P10	59	F	Low grade glial/glioneuronal tumor
P11	57	M	Glioblastoma (GBM), W.H.O. Grade IV
P12	52	M	Malignant spindle cell neoplasm
P13	51	F	Glioblastoma (GBM), W.H.O. Grade IV
P14	51	M	Glioblastoma (GBM), W.H.O. Grade IV
P15	38	M	Anaplastic astrocytoma, W.H.O. Grade III
P16	70	F	Glioblastoma (GBM), W.H.O. Grade IV
P17	23	M	Anaplastic astrocytoma, W.H.O. Grade III
P18	34	F	Diffuse astrocytoma, W.H.O. Grade II

**Table 2:** Summary results regarding tract-fMRI intersection for each functional region. The data in the table (X/Y) indicates that X of a total of Y activations are intersected by related AF (language) or CST (motor) tracts. 89 of 95 total activations are intersected by the related fiber tract. In some patients, multiple language tasks resulted in the identification of multiple putative Broca's or Wernicke's regions. All regions are included.

	Motor fMRI activations				Language fMRI activations	
	Foot	Hand	Finger	Lip	Broca	Wernicke
Left hemisphere	5/6	10/10	1/1	8/8	18/18	15/15
Right hemisphere	6/6	9/10	3/4	7/8	2/3	5/6

**Table 3:** Quantitative comparison of expert-selected and automatically identified tracts in the first 9 consecutive patients with brain tumors. Columns include the number of tracts identified or detected (ID), the mean and standard deviation of the tract volumes in cc, and the number of fMRI intersections, where patient-specific tracts were compared to patient-specific fMRI activations for language (AF) or motor (CST) tasks. The automatic identification produced significantly larger volumes for all structures (p < 0.01, paired t-tests).

	Expert selection			Automatic identification		
Tract	ID	Volume	fMRI	ID	Volume	fMRI
Left CST	9/9	17 ± 9	10/11	9/9	38 ± 15	11/11
Right CST	9/9	15 ± 5	11/11	9/9	41 ± 7	11/11
Left AF	9/9	31 ± 21	10/15	9/9	69 ± 32	15/15
Right AF	8/9	14 ± 16	1/4	9/9	38 ± 30	2/4

## Footnotes

1 HCP data are publicly available at https://db.humanconnectome.org/
